# F228. EFFECTIVENESS OF PALIPERIDONE PALMITATE VS OTHER LONG-ACTING INJECTABLE (LAI) ANTIPSYCHOTICS – AN ELECTRONIC CASE REGISTER STUDY

**DOI:** 10.1093/schbul/sby017.759

**Published:** 2018-04-01

**Authors:** Rashmi Patel, Edward Chesney, Matthew Taylor, David Taylor, Philip McGuire

**Affiliations:** 1 King’s College London; 2 Maudsley Hospital and Institute of Pharmaceutical Science, King’s College London

## Abstract

**Background:**

Paliperidone palmitate is a 2nd generation long-acting injectable (LAI) antipsychotic which is increasingly prescribed for patients with chronic schizophrenia. However, it is more expensive than 1st generation LAI antipsychotics and little is known about its effectiveness in a real world clinical setting. We sought to address this issue by analyzing a large electronic case register of patients with schizophrenia treated with LAI antipsychotics.

**Methods:**

Data were obtained from 1,281 patients in the South London and Maudsley NHS Foundation Trust (SLaM) Biomedical Research Centre (BRC) Case Register who were treated with an LAI antipsychotic between 1st April 2011 and 31st January 2015. The number of days spent as a psychiatric inpatient and the number of admissions to a psychiatric hospital were extracted using the Clinical Record Interactive Search tool (CRIS) and analyzed in each of the 3 years before and after LAI prescription using multivariable regression.

**Results:**

Patients who received paliperidone palmitate (n=430; 33.6%) spent more time in hospital (β coefficient 12.3 days, 95% CI 2.3 to 19.2, p=0.001) and were admitted to hospital more frequently (IRR 1.44, 95% CI 1.29 to 1.61, p<0.001) in the year prior to treatment than those treated with other LAI antipsychotics (n=851, 66.4%). However, there were no significant differences between paliperidone and the other LAI antipsychotics in the 3 years after initiation with respect to the number of days spent in hospital (β coefficient 5.4 days, 95% CI -57.3 to 68.2, p=0.86) or frequency of hospital admissions (Incidence rate ratio 1.07, 95% CI 0.62 to 1.83, p=0.82).

**Discussion:**

Paliperidone palmitate was more likely to be prescribed in patients with more severe illness, as indicated by a history of more frequent and lengthy hospital admissions prior to initiation. The absence of differences in outcomes after initiation indicates that the effectiveness of paliperidone palmitate was similar to that of other LAI antipsychotics. However, paliperidone palmitate may be better tolerated than other 1st generation LAI antipsychotics with a lower rate of discontinuation. These findings merit consideration in relation to the high cost of paliperidone palmitate compared to other LAI antipsychotics.

